# Female-selective mechanisms promoting migraine

**DOI:** 10.1186/s10194-024-01771-w

**Published:** 2024-04-24

**Authors:** Shagun Singh, Caroline M. Kopruszinski, Moe Watanabe, David W. Dodick, Edita Navratilova, Frank Porreca

**Affiliations:** 1 Banner - University Medicine Sunrise Primary Care, Tucson, AZ 85750 USA; 2 Department of Pharmacology, College of Medicine, University of Arizona, Tucson, AZ 85724 USA; 3 Department of Neurology, Mayo Clinic, Phoenix, AZ USA; 4 Atria Academy of Science and Medicine, New York, NY USA

**Keywords:** Pain, Migraine, Gepants, Calcitonin gene related peptide, Prolactin, Sexual dimorphism, Nociceptor

## Abstract

Sexual dimorphism has been revealed for many neurological disorders including chronic pain. Prelicinal studies and post-mortem analyses from male and female human donors reveal sexual dimorphism of nociceptors at transcript, protein and functional levels suggesting different mechanisms that may promote pain in men and women. Migraine is a common female-prevalent neurological disorder that is characterized by painful and debilitating headache. Prolactin is a neurohormone that circulates at higher levels in females and that has been implicated clinically in migraine. Prolactin sensitizes sensory neurons from female mice, non-human primates and humans revealing a female-selective pain mechanism that is conserved evolutionarily and likely translationally relevant. Prolactin produces female-selective migraine-like pain behaviors in rodents and enhances the release of calcitonin gene-related peptide (CGRP), a neurotransmitter that is causal in promoting migraine in many patients. CGRP, like prolactin, produces female-selective migraine-like pain behaviors. Consistent with these observations, publicly available clinical data indicate that small molecule CGRP-receptor antagonists are preferentially effective in treatment of acute migraine therapy in women. Collectively, these observations support the conclusion of qualitative sex differences promoting migraine pain providing the opportunity to tailor therapies based on patient sex for improved outcomes. Additionally, patient sex should be considered in design of clinical trials for migraine as well as for pain and reassessment of past trials may be warranted.

## Background

For reasons that are not well understood, women predominately suffer from chronic pain conditions including migraine, fibromyalgia, irritable bowel syndrome, temporomandibular disorder, rheumatoid arthritis, musculoskeletal disorders, complex regional pain syndrome and some forms of neuropathic pain [1–5]. Increased sensitivity to experimental pain has also been reported in healthy women [6]. Sex differences in pain may be considered as quantitative (i.e., prevalence) or qualitative in which patients of both sexes are diagnosed with the same disorder but the underlying mechanisms promoting pain are different [2, 3]. Qualitative sex differences in pain provide the opportunity to change current treatment paradigms by treating pain based on patient sex, a basic form of precision medicine.

In this review, we focus on two mechanisms that promote migraine and pain selectively in females and provide evidence supporting the conclusion that nociceptors, the fundamental building blocks of pain, are sexually dimorphic. Prolactin is a neurohormone that selectively sensitizes nociceptors from female animals or from female post-mortem human donors. Prolactin increases release of calcitonin gene-related peptide (CGRP), a neuropeptide that is causal in many people with migraine. Both prolactin and CGRP promote migraine-like pain behaviors selectively in female animals. Consistent with preclinical observations, small molecule CGRP-receptor (CGRP-R) antagonists (i.e., gepants) show preferential efficacy in acute migraine therapy in women [7, 8]. Sexual dimorphism therefore suggests that the uniform therapeutic approach for acute treatment of migraine, and pain-related disorders more generally, in men and women requires reassessment, and that opportunities exist for precision medicine based on patient sex.

## Introduction – migraine

Migraine is the 2nd leading cause of years lived with disability among both men and women of all ages and the 1st leading cause of years lived with disability among young women [9, 10]. This disorder affects more than 10% of adult population worldwide exceeding 1 billion people, with approximately 700 million being women [10, 11]. For reasons that are not understood, migraine is approximately three times more prevalent in women [12, 13]. Increased disability and medical care utilization has been observed among women with migraine as well as higher reported symptoms and elevated risk of migraine chronification [11, 14]. The high female prevalence of migraine suggests differential contribution of genetic influences and raises the possibility that mechanistic differences may exist in this disorder across sexes.

### Characteristics of migraine

Migraine is a multiphasic neurologic disorder that commonly includes a premonitory period, aura, headache, post-drome and interictal stages [15, 16]. A migraine attack can include both painful (i.e., unilateral throbbing headache, cephalic and extra-cephalic allodynia, neck pain) and non-painful symptoms such as visual disturbances, nausea, vomiting, fatigue, mood change, dizziness, enhanced sensitivity to touch (allodynia), light (photophobia), sound (phonophobia), smell (osmophobia) and cognitive dysfunction [17]. The migraine aura that occurs in approximately one-third of people with migraine, is thought to result from a transient wave of cortical depolarization [18–20]. Enhanced sensitivity to sensory stimuli likely results from mechanisms of sensory amplification including peripheral nociceptor sensitization, central sensitization and neuronal hyperexcitability. These features often, but not always, present in a time-sequence reflecting distinct, but sometimes overlapping, phases that characterize the complex migraine attack [16]. Collectively, these symptoms significantly affect ability to function, reduce quality of life [21–24] and impose a huge socio-economic burden [25–27]. The International Classification of Headache Disorders (ICHD-3) classifies migraine as episodic (EM) or chronic (CM) based on the clinical progression and frequency of episodes. Chronic migraine is defined as headache on more than 15 days a month for at least three months with at least 8 headache days each month meeting ICHD-3 criteria for migraine [17].

### Clinical treatment of migraine

Acute (attack-related) treatments for migraine used to treat migraine pain during an attack include over-the-counter drugs such as acetaminophen or non-steroidal anti-inflammatory drugs (NSAIDs) as well as migraine specific medicines such as triptans (i.e., sumatriptan, zolmitriptan, rizatriptan, naratriptan, frovatriptan and eletriptan) that act as agonists at the 5-HT1B/1D receptors and ditans (i.e., lasmitidan) that are agonists at the 5HT1F receptor [28–30]. Preventive treatments, designed to reduce the frequency, duration, and severity of attacks and generally initiated for people experiencing at least 4 headache days per month, have included non-specific beta blockers (e.g. propranolol, timolol and metoprolol), other antihypertensives (e.g.lisinopril and candesartan), antidepressants (e.g. pizotifen, amitriptyline, venlafaxine), anticonvulsants (e.g.valproate, gabapentin, topiramate), calcium channel blockers (verapamil, flunarizine), barbiturates (butalbital) and onabotulinumtoxinA for chronic migraine [28–30].

More recently, therapy for migraine has evolved to include drugs targeting the CGRP pathway that are used both for acute and preventive therapy [31]. Preventive therapies include monoclonal antibodies (mAbs) that sequester CGRP peptide (eptinezumab, fremanezumab, and galcanezumab) or block CGRP-R (erenumab). Small molecule antagonists of the CGRP-R, i.e., gepants, are used for acute migraine and include ubrogepant and rimegepant that are given orally and zavegepant that is administered intranasally. Atogepant (taken daily) and rimegepant (taken every other day) are gepant drugs that are also used for preventive therapy. CGRP targeting drugs have gained prominence and are often preferred over triptans for acute migraine treatment because of superior tolerability. Unlike triptans, CGRP-R antagonists do not produce vasoconstriction and are not associated with risks of cardiovascular adverse events. Moreover, CGRP targeting drugs are not associated with medication overuse headache (MOH) [28–30]. Despite female prevalence of migraine, with the exception of hormonal therapies, at present, migraine is treated with the same drugs in men and women.

### Sex differences in migraine

Female sex hormones have long been recognized as factors in promoting migraine across all life stages experienced by women [32–34]. The increased female prevalence of migraine begins at the age of menarche, peaks at age 40 and diminish after menopause [13, 35]. Many women experience increased migraine attacks at or around the time of their menstrual periods, in the first trimester of pregnancy and in the perimenopause. The onset of menstrual bleeding coincides with falling levels of hormones including progesterone and estrogen. The influence of gonadal hormones including especially estrogen has been a key focus of studies exploring sex differences in migraine. Female migraineurs show faster estrogen withdrawal during the late luteal phase than controls [36] and the number of migraine attacks has been found to be higher during the phases of falling estrogen and lower during the phases of rising estrogen [37]. These observations support the hypothesis that estrogen withdrawal along with changes in other hormones including oxytocin could trigger migraine attacks [38, 39].

Hormonal therapy consisting of estrogen replacement (i.e., contraceptives) are commonly used for women with migraine [40, 41]. Fluctuations in sex hormones have been shown to influence the release of CGRP and to activate the trigeminovascular system [42]. Women with regular menstrual cycles have higher CGRP concentrations in plasma and tear fluid [43]. Women using estrogen containing oral contraceptive pills have CGRP levels similar to women without migraine supporting the conclusion that stable estrogen levels may be protective for migraine possibly by influencing CGRP.

### Prolactin, CGRP and migraine

Recent work has also highlighted the role of prolactin, a circulating neurohormone that is released by the pituitary and that can also be produced locally by multiple cell types, in promoting migraine-like pain [34, 44, 45]. Significantly, this female predominant hormone enhances CGRP release [46]. Prolactin is under control of estrogen as well as stress. Women (and female animals) have higher circulating levels of prolactin than males and exhibit greater stress responses. Prolactin is released from pituitary lactotrophs and is under tight inhibitory control by hypothalamic dopaminergic tuberoinfundibular (TIDA) cells. Preclinical studies show that the TIDA cells are inhibited by stress-related transmitters resulting in increased circulating prolactin [47]. Dopamine D2 receptor agonists are used therapeutically to reduce release of prolactin from the pituitary. In humans, stress increases circulating prolactin, lowers sensory thresholds increasing the likelihood of pain attacks, is associated with painful menstruation (i.e., dysmenorrhea) and importantly, is the most common self-identified migraine trigger [39, 48]. Patients with very high levels of prolactin resulting from pituitary adenomas have increased migraine attacks that have been shown to respond to treatment by dopaminergic agonists that reduce prolactin levels [45, 49]. Estrogen and stress-related influences on prolactin are therefore likely to have a CGRP component in promoting migraine attacks in women.

### CGRP and prolactin are profoundly female-selective in their actions

Preclinical studies show that neurotransmitters can be sexually dimorphic in their effects. In preclinical models, CGRP produces female-selective pain and headache responses [50]. While rodent models cannot capture the complex multi-symptom and multi-phasic nature of migraine, they can and do provide mechanistic information that helps to explain migraine symptoms. As migraine headache likely arises from trigeminal nociceptors that innervate the cranial meninges, direct activation of these afferents in animals provides a surrogate measure of headache through assessment of pain behaviors including cutaneous cephalic allodynia, a translational measure that reflects a symptom that is also observed in patients during migraine attacks. Dural stimulants also elicit other surrogate measures including facial grimace in rodents that may be representative of ongoing headache pain. Importantly, application of CGRP directly to the dura mater elicited both evoked and ongoing migraine-like pain behaviors at much lower doses and with longer lasting effects in female, compared to male mice [50].

Reasons for enhanced CGRP-mediated pain responses in female animals remain unclear but could include sex differences in CGRP expression, distribution and signaling at receptors that bind this peptide. αCGRP, the primary neuronal form, is a potent 37-amino acid neuropeptide widely distributed in the trigeminovascular system including the trigeminal ganglion that is thought to be critical in promoting the headache phase of migraine through both neuronal actions and likely from dilation of meningeal blood vessels [51–56]. CGRP signals through the canonical CGRP-R and can also signal through the amylin 1 (AMY1) receptor. The canonical CGRP-R, consists of the calcitonin-like receptor (CLR), a G protein-coupled receptor, and an associated receptor activity modifying protein 1 (RAMP1) [53]. Additionally, CGRP can signal through the amylin 1 (AMY1) receptor that includes RAMP1 along with the G protein-coupled receptor, calcitonin receptor (CTR) [52]. In experimental human studies, activation of AMY1 which expresses RAMP1 but not CLR, can promote migraine in patients with primary headache disorders [53]. In contrast, adrenomedullin receptors 1 and 2 (AM1/2) that express CLR but not RAMP1 have not been implicated in inducing migraine. The female selectivity of CGRP might thus be influenced by sexual dimorphism in the expression of either CLR and/or RAMP1. At present, the relative contributions of these receptors to migraine continue to be an area of active investigation. Nevertheless, possible mechanistic insights may be deduced by a comparison of the relative affinities of the small molecule CGRP receptor antagonists (i.e., gepants) for these receptors. Ubrogepant, rimegepant and zavegepant all show comparable low picomolar affinities for the canonical CGRP receptor but vary significantly in their affinities for the AMY1 receptor [57]. In spite of this, the clinical benefits of gepants are very similar, suggesting that CGRP signaling promoting migraine likely requires interactions with both CLR and RAMP1. Preclinical studies demonstrated that while no sex difference is observed in CLR protein levels [58], administration of inflammatory mediators onto the dura mater of rodents induced increased mRNA expression of CGRP-R components in females when compared to males [59]. Additional studies in preclinical models and in human tissues are thus warranted and may help to clarify whether differences in expression of CGRP signaling components can contribute to female selective actions of CGRP and CGRP-R antagonists in migraine.

**Fig. 1:**
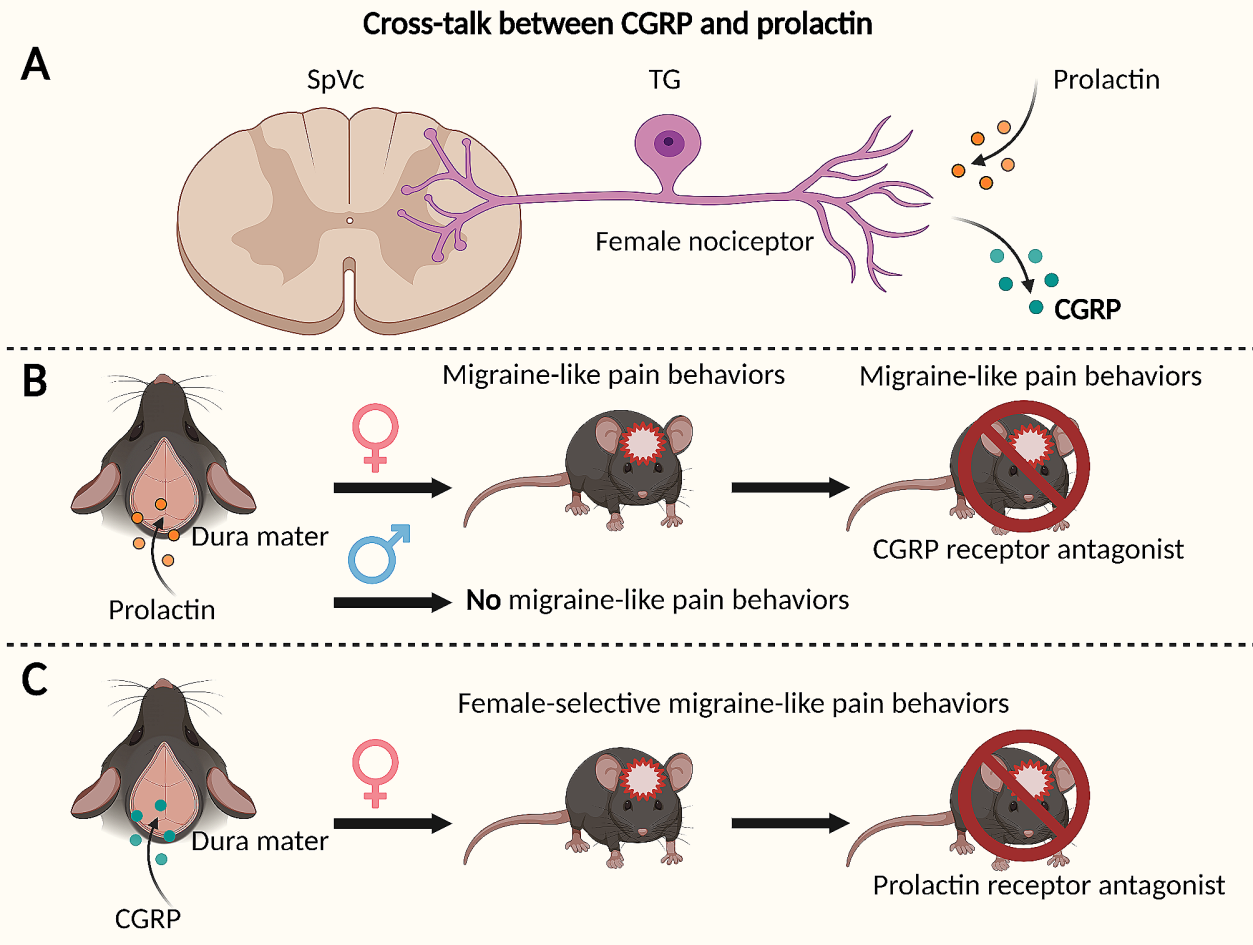
Cross-talk between prolactin and CGRP to produce migraine-like pain selective to females. **A** Increased CGRP release can be observed following prolactin sensitization of nociceptors selectively in females. **B** Application of prolactin to the mouse dura mater produces headache-like pain behaviors in female, but not male, mice. Prolactin-induced migraine-like pain behavior in females is blocked by a CGRP receptor antagonist [46]. **C** Application of CGRP to the mouse dura mater produces female-selective headache-like pain behavior which is blocked by a prolactin receptor antagonist [46]. PRL may be an upstream mechanism for CGRP-related migraine-like pain selectively in females

Preclinical studies have also consistently demonstrated that prolactin is selective in sensitizing female nociceptors and associated with increased release of CGRP [44, 60]. Application of prolactin to the mouse dura mater produces headache-like pain behaviors in female, but not male, mice that is blocked by either a prolactin receptor antagonist or by a CGRP receptor antagonist [44, 46]. These studies reveal cross-talk between prolactin and CGRP that is relevant to migraine-like pain in females (Fig. 1) [44, 46]. Stress-induced sensitization of trigeminal ganglion (TG) afferents to a transient receptor potential ankyrin 1 (TRPA1) agonist that has been linked to headache in humans has also been shown to be blocked by dopaminergic agonists or by interference with prolactin signaling at prolactin receptors selectively in female mice [47].

### Sexual dimorphism in rodent and human nociceptors

Recent discoveries have challenged our long-held understanding of somatosensory mechanisms promoting the perception of pain and headache. Nociceptors are sensory fibers with cell bodies residing in dorsal root ganglia (DRG) and TG that detect and transmit high intensity stimuli capable of eliciting actual or potential tissue damage from the peripheral tissues to the central nervous system [61]. Remarkably, analyses of human post-mortem DRG neurons have revealed that these cells are sexually dimorphic [62]. Differences in transcript, protein and function have emerged from analysis of sensory neurons of rodents, non-human primates and humans [62–66]. A key observation is sexual dimorphism in transcript for CGRP that is expressed at higher levels in DRG neurons recovered from human female, compared to male, donors [62]. Additionally, transcriptome analysis of non-neuronal satellite cells in post-mortem human DRG have also been found to differ between male and female tissues suggesting additional sexually dimorphic mechanisms that may promote neuronal activation and signaling [67, 68]. Most recently, sexual dimorphism has been observed in human nociceptors at the protein and functional level [65]. Thus, nociceptors, the fundamental building blocks of pain are different in men and women and, by extension, the mechanisms that may promote pain can also differ between the sexes.

Prolactin signals via homodimers of prolactin receptor (PRLR) long and short isoforms. PRLR homo dimers are composed of isoforms of the receptor. Prolactin (PRL) signaling at the long and short PRLR isoforms (PRLR-L and PRLR-S) have different effects. While signaling at homodimers of the PRLR long isoform activates intracellular gene transcription pathways, signaling at homodimers of the PRLR short isoform is pronociceptive by sensitizing effectors such as transient receptor potential (TRP) channels [47, 69–72]. Interestingly, PRLR heterodimers composed of two different receptor isoforms result in silencing of signaling [69, 73–75]. Thus, the balance of expression of the PRLR long and short isoforms determines the consequences of prolactin signaling. The female selectivity of PRL in promoting migraine-like pain behavior was confirmed by direct dural application in rodents [46]. PRL produced sustained and long-lasting migraine-like behavior in cycling and ovariectomized female, but not male rodents [46]. Consistent with this observation, PRLR are expressed at higher levels in DRG cells recovered from rodents, monkeys and from post-mortem female donors [65]. Furthermore, incubation of nociceptors from female, but not male, DRG cells including from human donors with prolactin produced increased excitability indicating dissociation of the properties of these cells [65].

Collectively, these studies support the conclusion that there are male and female nociceptors that can be distinguished at the transcript, protein and functional levels. While limited by assessment of a very small sample size, it appears that similar conclusions can be drawn from analysis of human TG neurons [76] Sexual dimorphism of nociceptors and the profound female selectivity of both CGRP and prolactin suggests an opportunity for the discovery and implementation of therapies for treatment of pain and migraine based on patient sex. Collectively, the growing body of evidence endorsing the functional distinction between “male” and “female” nociceptors might pave the way for an advanced understanding of mechanisms by which pain is produced in men and in women.

### CGRP is causal in promoting migraine in humans

Three crucial lines of evidence support the contribution of CGRP as a migraine substrate in humans: (a) increased levels of this transmitter were observed in the jugular outflow of people with migraine during attacks and normalization of CGRP levels was observed following relief of headache pain with sumatriptan [77]; (b) infusion of CGRP in people with migraine provokes headache with a migraine phenotype [54] and, as noted above, (c) therapies that prevent CGRP signaling are clinically effective. Critically, however, evidence supporting CGRP was obtained from studies conducted almost exclusively in women limiting conclusions regarding the causal role of CGRP across sexes. The initial study demonstrating elevated CGRP levels during migraine and normalization by sumatriptan was conducted in 8 patients, 7 of whom were women [77]. While all of the female patients showed elevated CGRP levels during migraine, responded to sumatriptan, and returned to baseline CGRP levels after treatment, the one male patient did not exhibit elevated CGRP during migraine, did not respond to sumatriptan and CGRP levels remained unchanged after treatment [77]. Thus, whether the conclusions of this seminal study may apply to men generally remains uncertain. Provocative studies of CGRP infusion to elicit migraine have also been conducted almost exclusively in women [78]. Such studies must be interpreted with caution as the pharmacological dose of CGRP used to elicit migraine may not reflect the contribution of endogenous CGRP in the trigeminal system during naturally occurring migraine. The clinical effectiveness of the gepants for acute migraine therapy provides the most convincing evidence for a causal role of CGRP in initiating a migraine attack.

### Sexual dimorphism in acute migraine therapy

The clinical effectiveness of the gepant drugs as well as CGRP-based antibodies strongly support the conclusion of a causal role of CGRP in migraine pathophysiology. However, CGRP-R antagonists are not effective in all patients for acute or preventive migraine treatment. Understanding which patient groups would preferentially respond to CGRP-R antagonists would improve patient care and avoid the use of these drugs in likely non-responders. An important consideration, therefore, is whether CGRP-R antagonists are effective in both men and women [7, 8].

The clinical and statistical reviews conducted by the FDA Center for Drug Evaluation and Research (CDER) concerning the New Drug Applications (NDA) for ubrogepant, rimegepant and zavegepant in migraine therapy are publicly available. These reviews supported the conclusion of efficacy in women based on a sex-specific analysis of pooled pivotal trial data for both CGRP-R antagonists effects on the co-primary endpoints of pain freedom (PF) and absence of the most bothersome symptom (MBS) at the 2-hour time point [73, 74]. However, there was no evidence of efficacy for acute migraine treatment with these drugs in men. For example, ubrogepant demonstrated a treatment benefit effect over placebo of 8.3% for PF and 12.4% for MBS in women while effect over placebo was 0.2% for PF and 0.7% for MBS in men [79]. Similarly, the treatment benefit effect of rimegepant over placebo in women was 8.9% for PF and 10.2% for MBS, while in men, the rimegepant benefit effect over placebo was 1.1% for PF and 6.6% for MBS [80]. These findings highlight the sex-based differences in the impact of CGRP-related migraine treatments and reveal that CGRP-R antagonists demonstrate significant therapeutic benefit in the acute treatment of migraine in women but do not provide evidence of efficacy in men [7, 8].

The reasons for the apparent enhanced efficacy of CGRP-R antagonists for acute migraine in women remain unclear. The current absence of therapeutic benefit in men might be attributed to potential limitations in statistical power as most clinical trials including those with ubrogepant and rimegepant were comprised predominately of women. Other possibilities may also be considered. For example, women might treat attacks earlier resulting in improved effects. However, all patients are required to wait until pain is at least moderate before administering study drug. Also, improved outcomes are not seen across a range of other therapies including triptans [33]. Based on currently available data, it is not possible to conclude that these drugs provide clinical benefit for acute migraine treatment in men. It remains to be determined if this conclusion is supported by future studies. Nevertheless, the female selectivity of CGRP in promoting migraine-like pain and the apparent female selective effects of CGRP-R antagonists support the possibility that fundamental biological differences exist between men and women in mechanisms of migraine, and other, pain conditions.

Data from the CDER reviews of gepant drugs are also consistent with the likely influence of female hormones across life. Possibly diminished efficacy in promoting PF from acute migraine at the 2-hour timepoint after treatment with ubrogepant and rimegepant was observed in a small number of patients over age 65. While the reported data from these trials were not separated by sex, both trials included a high percentage of women (approximately 85%) suggesting that the decreased effect observed in older patients may represent the contributions of a large group of post-menopausal women. Similarly, recent CDER evaluation of zavegepant data reveal diminished effects when studied in patients over the age of 40 [81]. In patients younger than 40, zavegepant was significantly more effective in producing pain freedom at 2 h than in those over age 40 [81]. This trial also did not separate the data by sex but the population over the age of 40 likely includes a larger proportion of post-menopausal women than those under 40 [81]. Possible differences may thus occur in the efficacy of CGRP-R antagonists in postmenopausal women who generally have lower CGRP levels [43]. The smaller effect observed in older patients is therefore consistent with diminished effects of CGRP-R antagonism in postmenopausal women.

## Concluding remarks and future perspectives

Despite increasing evidence of sexual dimorphism in neurological diseases, patient sex is usually not considered in the choice of therapies for migraine and other pain conditions. The great majority of historical preclinical studies on pain and migraine have exclusively used male rodents [3, 82]. In the past decades, the funding agencies mandate policies requiring the incorporation of sex as a variable in preclinical research [82]. This mandate has led to important findings revealing sexual dimorphism in human and animal nociceptors, suggesting the significant sex-related distinction in migraine and pain pathophysiology. This emerging perspective underscores the need to factor in patient sex when determining the most appropriate therapeutic approach and to address potential qualitative sex differences in neurological disorders. Information from preclinical studies appear to align well with clinical data and supports the conclusion of a qualitative sex difference in migraine pathophysiology related to female selectivity of prolactin and CGRP. The relative absence of data demonstrating clinical benefits of CGRP-R antagonists for acute migraine treatment in men suggests the need for a careful consideration of decision making in the choice of care and for discussion and patient disclosure. Additionally, to establish efficacy in men, dedicated and appropriately powered clinical trials should be performed. Similarly, efficacy estimates should be established for pre- and postmenopausal women separately. Consequently, addressing the unmet medical need for satisfactory migraine therapy in men calls for approaches that extend beyond CGRP-based mechanisms. The female selective role of prolactin in sensitization of trigeminal nociceptors suggest a therapeutic opportunity for development of novel prolactin targeting therapies including anti-prolactin antibodies [83].

These findings with migraine also raise the possibility that conclusions as to the efficacy of CGRP-based therapies for other, non-migraine, but female prevalent pain conditions such as fibromyalgia, irritable bowel syndrome and others could have been influenced by the relative proportion of men and women, as well as the percentage of pre- vs. postmenopausal women, in any given trial. Overall, our conceptual view of therapy for migraine and other pain conditions requires adjustment so that studies consider patient sex as the most fundamental aspect of precision medicine. The future direction in the field of migraine management should prioritize personalized treatment strategies that take into account the sex-related differences in pathophysiology, thus paving the way for more effective and tailored care for individuals suffering from migraine.

## Data Availability

No datasets were generated or analysed during the current study.

## Abbreviations

AM1: adrenomedullin receptors 1
AM2: adrenomedullin receptors 2
AMY1: amylin 1 receptor
CDER: Center for Drug Evaluation and Research
CGRP: calcitonin gene-related peptide
CGRP-R: CGRP-receptor
CLR: calcitonin-like receptor
CM: chronic migraine
DRG: dorsal root ganglia
EM: episodic migraine
ICHD-3: International Classification of Headache Disorders
NSAIDs: non-steroidal anti-inflammatory drugs
mAbs: monoclonal antibodies
MBS: most bothersome symptom
MOH: medication overuse headache
NDA: New Drug Applications
PF: pain freedom
PRL: prolactin
PRLR: prolactin receptor
PRLR-L: prolactin receptor long isoform
PRLR-S: prolactin receptor short isoform
RAMP1: receptor activity modifying protein 1
TIDA: dopaminergic tuberoinfundibular
TG: trigeminal ganglion
TRP: transient receptor potential
TRPA1: transient receptor potential ankyrin 1
